# A Novel Case of Providencia rettgeri Osteomyelitis Presenting in the Frontal Bone

**DOI:** 10.7759/cureus.43367

**Published:** 2023-08-12

**Authors:** Sydney Grubb, Keana-Kelley D Swanner, Carlos Cebollero

**Affiliations:** 1 Critical Care, Alabama College of Osteopathic Medicine, Tallahassee, USA; 2 Medical School, Alabama College of Osteopathic Medicine, Dothan, USA; 3 Hospitalist, Tallahassee Memorial Hospital, Tallahassee, USA

**Keywords:** frontal bone osteomyelitis, neurosurgery, providencia rettgeri, osteomyelitis treatment, osteomyelitis

## Abstract

Osteomyelitis of the skull is a particularly life-threatening condition. Infections are usually at the base of the skull and typically occur following dissemination from another site, such as the external auditory canal. Typical organisms include *Pseudomonas* and *Staphylococcus* species. This paper demonstrates an unusual case of osteomyelitis of the frontoparietal bone, as well as the first published case of *Providencia rettgeri *causing cranial osteomyelitis in humans.

## Introduction

Osteomyelitis of the skull is an uncommon but life-threatening condition. Typically, the infection occurs at the base of the skull following another pathologic infection, such as malignant otitis externa [1]. Certain patients can also get the condition following neurosurgical procedures. Here, we present an unusual case of a patient with osteomyelitis of the frontoparietal bone, caused by *Providencia rettgeri*, *Proteus mirabilis*, and methicillin-sensitive *Staphylococcus aureus*. To the authors’ knowledge, this is the first published case of *Providencia rettgeri* causing osteomyelitis in the cranium in humans.

## Case presentation

A 62-year-old Caucasian male presented to an outside emergency department for evaluation of syncope. The patient had a past medical history of chronic alcoholism, and a post-traumatic subdural hematoma six months prior which required craniotomy and evacuation. Following the craniotomy, the patient was unable to follow up with his previous neurosurgeon due to the surgeon’s unfortunate passing. In the outside emergency department, a CT of the head showed an acute-on-chronic subdural hematoma. Following the standard of care guidelines, he was started on broad-spectrum antibiotics (vancomycin) and transferred to our facility.

**Figure 1:**
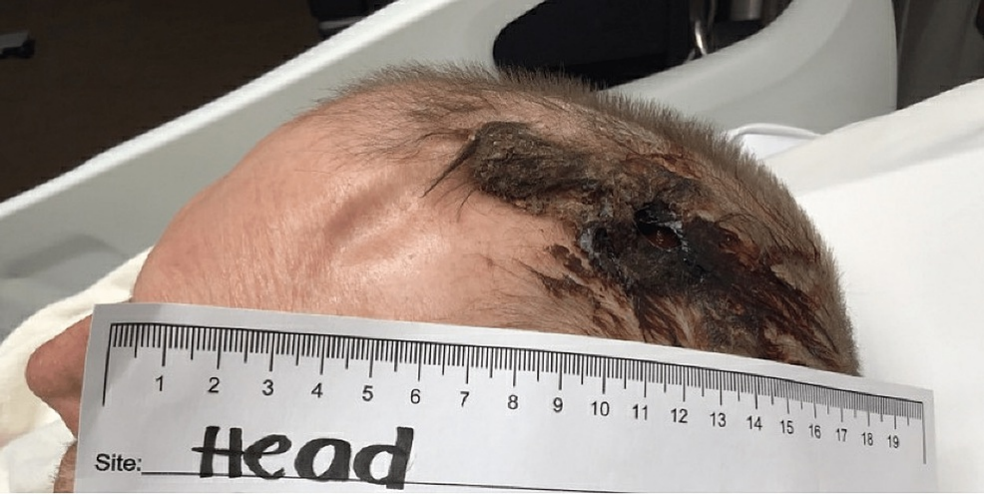
Presenting wound. A 2 cm hole in the left parietal scalp with purulent drainage and visible underlying brain matter.

Upon arrival, the physical exam showed a 2 cm hole in the left parietal scalp with purulent drainage and underlying visible brain matter (Figure 1). The tissue showed necrotic edges and smelled of decay. The patient was hemodynamically stable and neurologically intact at this time.

Notable lab results included sodium of 119 mmol/L (likely alcohol-related hyponatremia), white blood count of 5.1 × 1,000/mm^3^, hemoglobin of 11.2 g/dL, alcohol level <10 mg/dL, C-reactive protein of 0.42 mg/dL, and a negative urinalysis.

**Figure 2:**
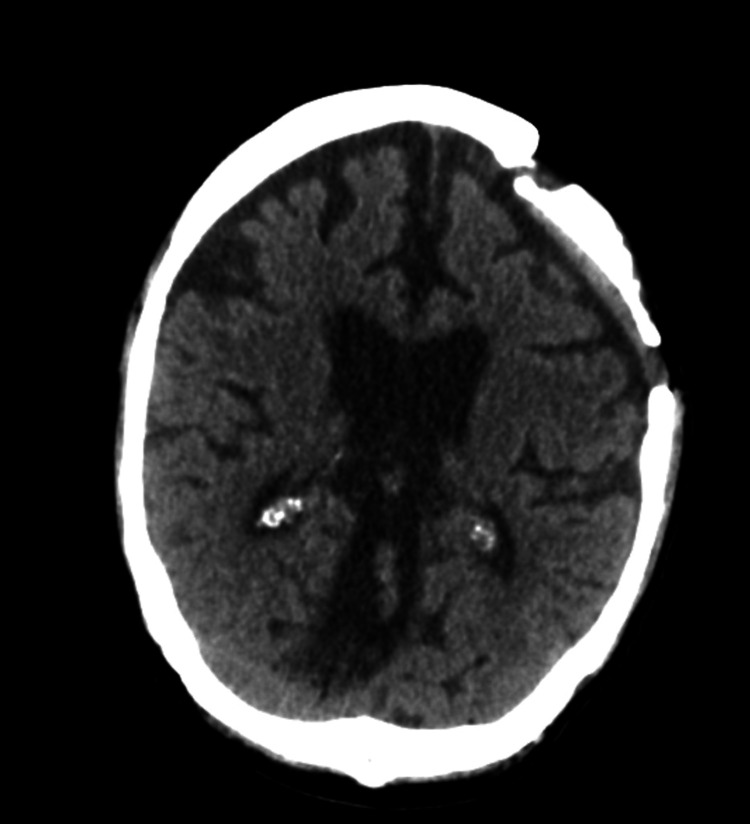
CT of the brain without contrast, initial, showing severe osteomyelitis of the left frontoparietal bone.

A repeat CT of the head showed a “mixed-density subdural hematoma, as well as a severe irregularity of the craniostomy flap with apparent ulceration of the scalp along the superior margin of the craniostomy. Findings could be related to osteonecrosis or chronic osteomyelitis” (Figure 2).

On the first night of admission, the patient had an episode of non-sustained ventricular tachycardia, then polymorphic ventricular tachycardia thought to be secondary to the prolonged QTc from the acute head bleed. The patient was administered 2 g of magnesium because he was hypomagnesemic at 1.7. Beta-blockers were unable to be administered due to the patient’s hypotension. The patient was started on vasopressin 6 mL per hour for hypovolemic shock. There was also speculation of possible secondary adrenal insufficiency, so the patient was started on hydrocortisone IV 50 mg every six hours. Any of these could have contributed to the patient’s episode of ventricular tachycardia, and no further episodes were reported throughout his stay.

The patient was started on broad-spectrum IV vancomycin 1 g every 12 hours and IV cefepime 2 g every six hours. Preliminary gram stain showed *Staphylococcus aureus* and blood cultures were negative. IV metronidazole was added 0.5 g (100 mL) every six hours.

**Figure 3:**
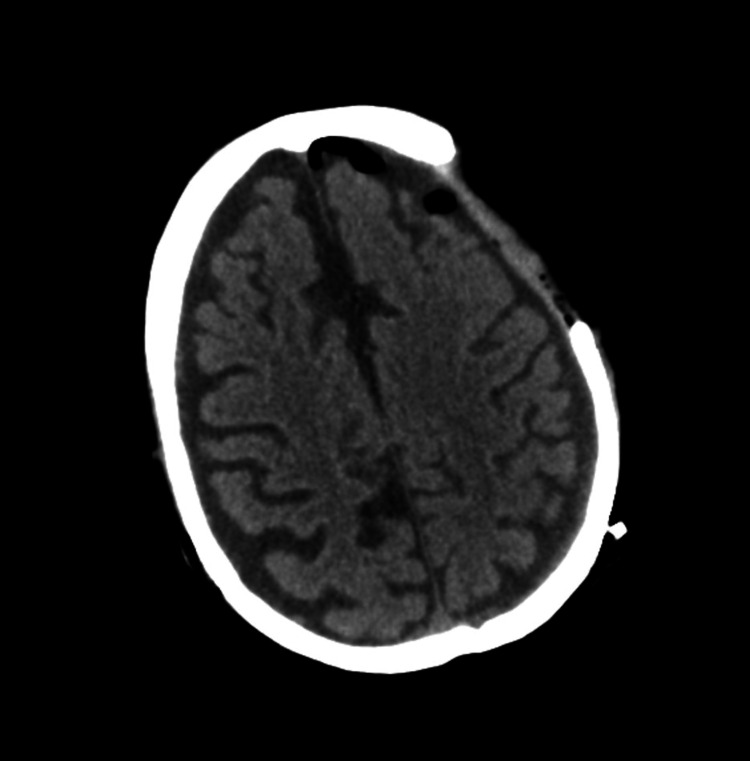
CT of the brain without contrast, post-craniectomy, showing a large area of removed bone and areas of pneumocephalus.

On hospital day seven, the patient underwent a craniectomy with debridement of the skull and soft tissue for cranial osteomyelitis with wound dehiscence. Post-procedure CT still showed low-density left hemisphere subdural collection at 0.9 cm in max thickness and post-surgical pneumocephalus. There was no midline shift or herniation (Figure 3). At this time, the patient was comfortable and able to follow commands.

Wound cultures grew *Providencia rettgeri*, *Proteus vulgaris*, and methicillin-sensitive *Staphylococcus aureus*. Vasopressin was discontinued, and shock was deemed likely septic with some component of hyponatremia.

The patient was transferred out of the intensive care unit two days following the procedure with a plan for six weeks of IV cefepime 2 g every 12 hours and metronidazole 1 g (200 mL) every 12 hours, as well as a possible cranioplasty outpatient.

The patient continued to receive daily antibiotics. He complained of continued severe pain and began attempting to pull out his IV and requesting to leave against medical advice. Multiple discussions were held with the patient, the patient’s family, the medical team, and hospice/palliative care. After 20 days in the hospital, the patient elected to discontinue treatment and was admitted to hospice care.

## Discussion

The genus *Providencia* is a biofilm-producing, urease-producing, gram-negative bacillus of the family Enterobacteriaceae and includes *Providencia stuartii*, *P. rettgeri*, *P. alcalifaciens*, *P. rustigianii*, and *P. heimbachae*. Providencia species are non-lactose-fermenting, mannitol-fermenting, and able to use citrate. *P. rettgeri* and *P. stuartii* are commonly found in water, soil, and animal reservoirs, and are opportunistic pathogens in hospitalized patients and elderly residents in a nursing care facility. *Providencia *tends to be opportunistic and infections are often polymicrobial. Due to these factors, it tends to be a multidrug-resistant organism [2]. Due to the emergence of commonality within the geriatric and nursing home populations associated with indwelling-catheter use, the genus is likely to become more drug-resistant in the future [3].

*P. rettgeri* is an opportunistic pathogen that has been previously seen in nosocomial infections, including catheter-induced urinary tract infections, bacteremia, skin infections, ventilator-associated pneumonia, ocular infections, meningitis, and gastroenteritis. *P. rettgeri* has been seen most commonly in geriatric and immunocompromised patients. The urease-producing factors lead it to become a common co-infection of *Proteus mirabilis* within the urinary tract of patients who have long-term indwelling catheters. The biofilm production causes the bacteria to be intrinsically resistant to antibiotics, which increases the probable mortality among the patients, as well as increases the financial burden on the patients and the hospital. *P. stuartii* and *P. rettgeri* are also etiologic isolates of purple urine bag syndrome, characterized by the purple color of the indwelling urinary catheter [3,4].

In neurosurgical cases, postoperative infections are a major cause of morbidity and mortality. Gram-positive organisms such as *Staphylococcus aureus* and polymicrobial flora are usually implicated. Previous neurosurgical cases of *P. rettgeri* described colonization within brain catheters as an emerging rare post-surgical complication [2]. There was a single published case of *P. rettgeri* osteomyelitis in a rattlesnake in 2002, but the snake ended up dying before the study concluded [5]. Cranial osteomyelitis by *P. rettgeri* is yet to be reported in the literature to the author’s current knowledge and extensive search.

Management

*P. rettgeri* is known to be intrinsically resistant to multiple drugs, including first-generation cephalosporins and polymixins. However, infections are becoming increasingly more difficult to treat due to newly isolated strains that produce carbapenemases [6]. The bacteria can additionally form biofilms, which further limits antibiotic penetrance. Culture and sensitivity tests are often done in infections that prove persistent despite treatment. Treatment varies depending on these results, but have been successfully treated with clindamycin, fluoroquinolones, aminoglycosides, and later-generation cephalosporins [2].

Physician death

As stated earlier, this unfortunate case arose because the patient’s neurosurgeon passed away and he was unable to follow up with a new neurosurgeon. With the aging population in the United States, the death of older professionals is a scenario society needs to be prepared for. While there are guidelines describing how facilities should handle physician deaths, there is no standardized process or legal standard for distributing patients to other facilities or finding new providers for patients. In many cases, the duty to find a new physician may fall to the patients themselves [7]. Perhaps more guidelines need to be created for how private practices and other healthcare organizations care for patients following an unfortunate passing. As illustrated above, the lack of follow-up for patients can be life-threatening.

## Conclusions

This case report represents the first published case of *P. rettgeri* causing cranial osteomyelitis in humans. Due to the inherent antimicrobial resistance and the emergence of new resistance to carbapenems seen in some strains, it is important for early diagnosis, culture and sensitivity testing, and treatments to avoid outbreaks that have been seen previously in some hospitals throughout Asia. Additionally, this case highlights the need for early diagnosis and treatment of cranial osteomyelitis, as cases can rapidly become fatal. Finally, this case highlights the need for plans to be put in place for when physicians pass away and for who is to take over patient care. As demonstrated, in these unfortunate situations, patients can fall through the cracks, leading to serious and deadly outcomes.

## References

[1] Sokołowski J, Lachowska M, Karchier E, Bartoszewicz R, Niemczyk K (2019). Skull base osteomyelitis: factors implicating clinical outcome. Acta Neurol Belg.

[2] Sapkota S, Karn M, Regmi SM, Thapa S, Miya FU, Yonghang S (2021). Providencia rettgeri infection complicating cranial surgery: illustrative cases. J Neurosurg Case Lessons.

[3] Wie SH (2015). Clinical significance of Providencia bacteremia or bacteriuria. Korean J Intern Med.

[4] Washington MA, Barnhill J, Griffin JM (2015). A case of wound infection with Providencia rettgeri and coincident gout in a patient from Guam. Hawaii J Med Public Health.

[5] Ramsay EC, Daniel GB, Tryon BW, Merryman JI, Morris PJ, Bemis DA (2002). Osteomyelitis associated with Salmonella enterica SS arizonae in a colony of ridgenose rattlesnakes (Crotalus willardi). J Zoo Wildl Med.

[6] Shin S, Jeong SH, Lee H, Hong JS, Park MJ, Song W (2018). Emergence of multidrug-resistant Providencia rettgeri isolates co-producing NDM-1 carbapenemase and PER-1 extended-spectrum β-lactamase causing a first outbreak in Korea. Ann Clin Microbiol Antimicrob.

[7] Tessier W, Keegan W, Ash E (2018). Continuing obligations following the unexpected death of a physician: things to keep in mind. Mo Med.

